# What Percentage of Patients is a Candidate for Unicompartmental Knee Replacement at a Chinese Arthroplasty Center?

**DOI:** 10.2174/1874325001812010017

**Published:** 2018-01-30

**Authors:** Yong He, Lianbo Xiao, Weitao Zhai, Maximilian F. Kasparek, Guilin Ouyang, Friedrich Boettner

**Affiliations:** 1 Department of Orthopedic Surgery, Shanghai Guanghua Hospital, No.540 Xinhua Road, 200052, Shanghai, China; 2 Department of Orthopedics, Vienna General Hospital, Medical University of Vienna, Waehringer Guertel 18-20, 1090 Vienna, Austria; 3 Adult Reconstruction & Joint Replacement Division, Hospital for Special Surgery, 535 East 70^th^ Street, New York, NY 10021, USA

**Keywords:** Osteoarthritis, Indication, Unicompartmental knee arthroplasty, Chinese population, Kozinn criteria, Oxford criteria

## Abstract

**Background:**

Data on indication of Unicompartmental Knee Arthroplasty (UKA) in the Asian population are currently not available. The current paper evaluates patients undergoing knee replacement at a Chinese Orthopaedic Specialty Hospital to report the percentage of patients who meet radiographic and clinical indication criteria for UKA.

**Methods:**

Over a one-year period 463 consecutive patients (515 knees) underwent primary knee replacement surgery. Clinical data were recorded and preoperative radiographs were assessed. Patients were classified as suitable candidates for UKA based on the degree of deformity, preoperative ROM and radiographic appearance of osteoarthritis. The different indication criteria for body weight and extend of patellofemoral osteoarthritis as reported by Kozinn and Scott as well as the Oxford Group were applied.

**Results:**

160 knees (31%) were excluded because of inflammatory and posttraumatic arthritis. 55 knees had to be excluded because of incomplete radiographs. Of the remaining 300 knees with osteoarthritis, 241 knees were excluded because of extend of deformity (n=156), decreased range of motion (n=119), advanced patellofemoral arthritis with bone loss (n=11) and AP instability (n=1). Of the remaining 63 knees, 54 knees (18%) met the modified Oxford criteria for mobile UKA and only 25 knees (8%) met the Scott and Kozinn criteria for fixed UKA.

**Conclusion:**

The current paper suggests that in comparison to Caucasian population, only a smaller percentage of patients at a Chinese Orthopaedic Specialty Hospital meet the indication criteria for UKA. Therefore, it might make sense to concentrate UKA surgeries in high volume centers.

## 1 INTRODUCTION

Unicompartmental Knee Arthroplasty (UKA) is a valuable treatment option for patients with unicompartmental osteoarthritis (OA) [1-5]. Patient selection is the key to assure excellent clinical outcomes of UKA. If performed for the right indication UKA increases patient satisfaction and functional outcomes compared to total knee arthroplasty (TKA) [6].

Previous reports have estimated that up to 47.6% of all patients, who undergo knee arthroplasty in the United Kingdom (UK) are possible candidates for UKA [7].

Cohort studies between Chinese and Caucasians identified interracial differences in prevalence and severity of osteoarthritis [8]. OA is more common in older Chinese female patients compared to Caucasians [8] and prevalence of lateral OA is higher in Chinese [9]. Moreover, valgus alignment of the distal femur is more common in Chinese [10]. Despite a lower BMI Asians present for surgery at a younger age with greater pain and dysfunction [11].

Despite these interracial differences in OA, data on the indications of UKA in the Chinese population are currently not available. Therefore, the current paper analyses: What percentage of patients undergoing knee arthroplasty in an Orthopaedic Specialty Hospital in China meet the indication criteria for unicompartmental knee arthroplasty based on radiographic appearance, degree of deformity and preoperative range of motion.

## 2 MATERIAL AND METHODS

All patients undergoing primary knee arthroplasty at a Chinese specialized orthopedic department in 2014 were evaluated in the current study. The study was approved by the institutional review board at the author’s institution.

In total 463 patients who underwent 515 primary knee arthroplasty were evaluated. Patient demographics including age, BMI and clinical data on ROM were collected retrospectively. Patients who had surgery due to rheumatoid arthritis (146 knees), gout arthritis (8 knees), posttraumatic arthritis (3 knees), bone tumor (1 knee) and ankylosing spondylitis (2 knees) were excluded in the first step. After exclusion of 55 knees with incomplete radiographs, 300 knees with osteoarthritis were considered for further radiological assessment. Standard preoperative radiographs, including standardized weight-bearing long standing hip to ankle radiographs, anterior-posterior weight bearing (AP), lateral and patella merchant view radiographs were available for evaluation. An orthopedic surgeon reviewed all preoperative radiographs. Limb alignment was measured as previously described by Cook [12]. Berend **et al*.* [13] reported that coronal axis deviation of more than 10° and more than 15 degree flexion contracture suggest ACL (anterior cruciate ligament) deficiency. Therefore, patients with more severe axis deviation were not considered UKA candidates.

The lateral radiographs were assessed for disease location to assess ACL (anterior cruciate ligament) function according to the modified Keyes classification [14]. The degree of OA in the lateral compartment was assessed using the Kellgren and Lawrence grading [15]. The extend of patella femoral OA was assessed using the Ahlbäck [16] radiographic grading scale. The assessment of lateral osteophytes used the OARSI atlas, which was published by Altman and Gold [17].

**Fig. (1):**
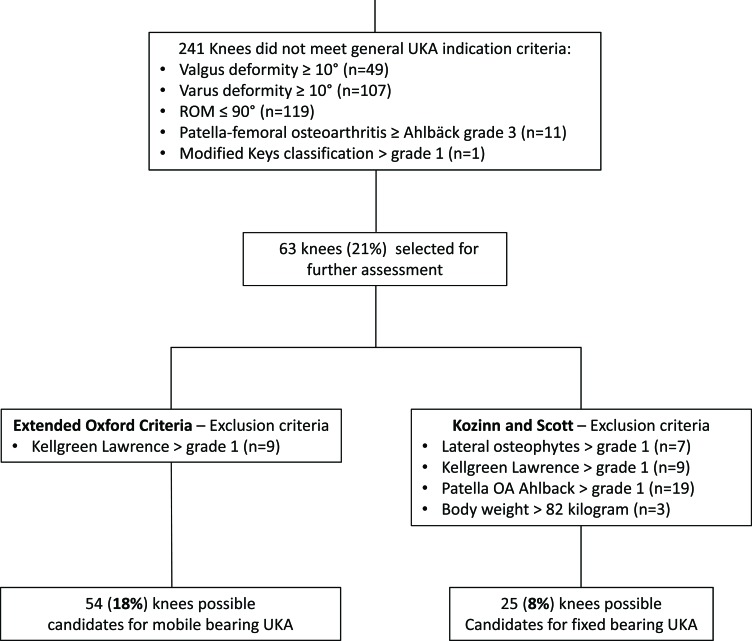
The flowchart shows all exclusion and inclusion criteria of the 300 analyzed patients.

The indication criteria are based on the “Consensus Statement on Indications and Contraindications for Medial Unicompartmental Knee Arthroplasty“ criteria pubished by Berend *et al*. [13]. Moreover, the radiological assessment was comparable to the decision aid for UKA published by the Oxford group [18] except that varus and valgus stress radiographs were not available. The radiologic assessment was performed in two steps. During the first step patients who meet general UKA inclusion criteria like valgus or varus deformity of less than 10 degrees, range of motion of more than 90 degrees, flexion contracture of no more than 10 degrees, and up to grade 2 patella femoral OA (Ahlbäck grading) were identified for further assessment (Fig. **1**).

**Fig. (2):**
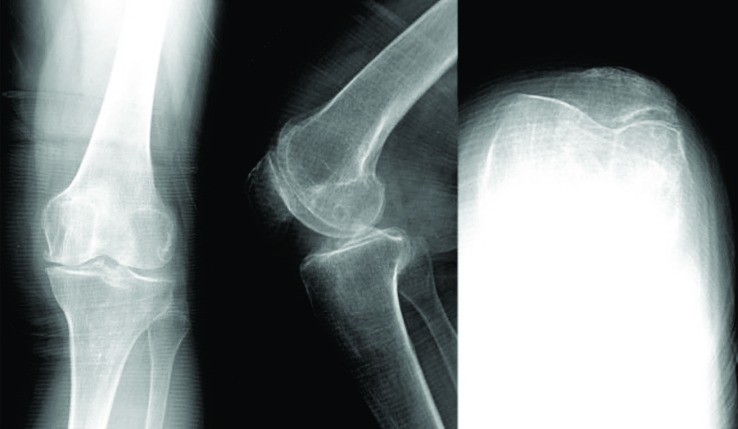
A patient with medial osteoarthritis with intact ACL and moderate retropatellar arthritis is shown. This patient was considered a suitable candidate for UKA according to the modified Oxford criteria.

In the second step, the specific criteria described by Kozinn and Scott [19] and the more extended criteria described by the Oxford group [18] were applied to identify candidates for UKA. According to the Kozinn and Scott [19] criteria only patients with Ahlbäck grade one patellofemoral OA, a modified Keys grade 1, lateral osteophytes grade 1 and Kellgren and Lawrence grade 0 and 1 in the lateral compartment were considered candidates for UKA (Fig. **2**).

**Fig. (3):**
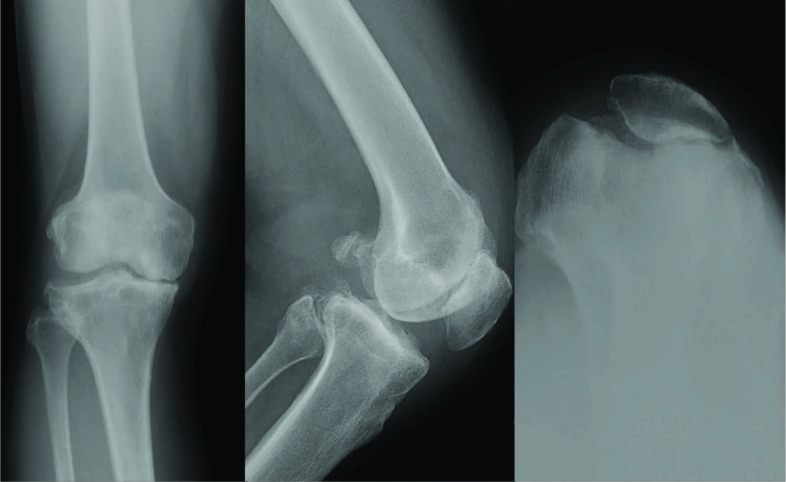
The radiographs present a patient, who is not a suitable candidate for UKA due to bone on bone retropatellar osteoarthritis despite medial osteoarthritis and intact ACL.

In addtion for the Oxford group, we included patients with Ahlbäck grade 2 in the patellofemoral compartment and patients with lateral osteophytes grade 2 (Fig. **1**). Therefore, only patients with evidence of bone loss on the patella (Ahlbäck grade 3-5) and patients with Kellgren Lawrence grade 2 and 3 lateral joint space changes were excluded in the modified Oxford group in accordance with the Oxford Decision Aid Flyer [18] (Fig. **3**).

Of the patients that met the indication all were at least 60 years old and therefore no patient was excluded because of age or high activity level (Kozinn and Scott criteria). Patients with more than 82 kg body weight were excluded according to the Kozinn and Scott criteria but were considered candidates according to the modified Oxford criteria.

## 3 RESULTS

463 patients who underwent 515 knee arthroplasty, were included in the current study. 160 knees were excluded because of inflammatory arthritis, posttraumatic arthritis and bone tumors (31%). 55 knees had to be excluded because of missing radiographs leaving 300 knees with osteoarthritis of the knee for evaluation.

The average age of the patients was 71 years (range 52 to 90 years). The average BMI was 26.7 (range 17 to 39) and average preoperative ROM was 96.5° (range 5 to 140). 119 knees showed 90 degrees of flexion or less. 122 knees presented with a flexion contracture (average 12.6 degrees; range 5 to 45). The average mechanical alignment of patients with a varus and valgus deformity was 9.2 degrees (range 0° to 28.2°) and 5.2 degrees (range 0.8° to 27.8°) respectively.

Of the 300 patients with osteoarthritis in a first step patients that did not meet general indication criteria for UKA were excluded: 119 knees were excluded because of poor preoperative range of motion, 156 knees because of increased mechanical deformity, 11 knees had grade 3 or higher patellofemoral arthritis and one knee had a grade 2 in the modified Keys classification. Since some knees met multiple exclusion criteria a total of 237 knees were excluded (Fig. **1**).

The remaining 63 (21%) knees were assessed using the criteria established by Kozinn and Scott as well as the Oxford criteria. According to the Kozinn and Scott criteria: Seven patients had more than a grade 1 osteophyte and 9 patients had arthritis beyond Kellgren Lawrence grade 1 in the opposite compartment leaving 47 patients. For the Kozinn and Scott criteria an additional 19 patients were excluded with more than grade 1 Ahlbäck changes in the patellofemoral compartment and 3 patients were excluded because of more than 82-kilogram body weight. Overall 25 out of the 300 knees (8%) met the criteria for a UKA set by Kozinn and Scott. One of the patients had valgus alignment.

According to the Oxford criteria, no patient had more than a grade 2 osteophytes and 9 patients had arthritis beyond Kellgren Lawrence grade 1 in the opposite compartment. Therefore 54 patients (18%) were identified as suitable UKA candidates of which one patient had valgus alignment.

## 4 DISCUSSION

The current study suggests that a smaller percentage of Chinese patients undergoing knee arthroplasty are candidates for UKA. Out of 300 knees with osteoarthritis only 18% met the modified Oxford criteria and only 8% met the Kozinn and Scott criteria. The radiological assessment was comparable to the described decision aid by Hamilton *et al*. [20], which has a high sensitivity and specify to identify suitable candidates for UKA. The decision to include patients in the UKA cohort was performed independently without any preference for UKA. Patients that meet the radiological criteria for UKA have 99% survival rate within 5 years supporting the current patient selection [20].

Limitations of the current study are [1] that only radiographs were assessed and MRI imaging and intraoperative assessment of the opposite, patellofemoral compartment or ACL were not available [2]. Stress radiographs were not available, however Waldstein *et al*. [21] reported that valgus stress radiographs provide no additional diagnostic information in lateral cartilage assessment in comparison to AP radiographs [3]. Data on Caucasian patients were derived from the literature and there was no matched comparative group in the current study [4]. These are data from an orthopaedic specialty hospital and it could be that this resulted in a selection bias for patients with more severe deformities and stiffness [5]. Cultural and socioeconomic factors patients might seek joint replacement care at a later time point and the percentage of patients meeting indication criteria for UKA might change should these factors change in the future.

More recent studies on US patients suggest that 12 to 26% of the patients might qualify for UKA according to the Kozinn and Scott critieria [22]. These numbers are substantially higher than the percentage reported in the current study. In a prior study by Stern and Insall [23] in 1992, they prospectively evaluated 228 knees according the Kozinn and Scott criteria. According to this paper 13 knees (6%) were suitable candidates for UKA and the authors concluded that with proper patient selection the number of UKAs becomes relatively small. However, the majority of patients (85%) were not excluded because of objective clinical or radiographic findings but rather because of the subjective intraoperative assessment. Pandit [2] and Price [24] extended the UKA criteria for the Oxford UKA system since their data showed that weight, age, activity level and the state of the patellofemoral joint did not have a negative impact on short-term outcome. A suitable candidate for UKA should have predominantly medial osteoarthritis, a preserved lateral joint space, a functionally intact anterior cruciate ligament as well as a correctable deformity. Murray [4] suggested that these criteria were satisfied in about 50% of knees requiring arthroplasty, and that a much larger population can be considered as a candidate for UKA. Willis-Owen and coworker [7] examined radiographs of 200 consecutive knees who were scheduled for knee arthroplasty and concluded that 91 knees (47.6%) were suitable candidates for UKA. In addition, the authors considered 60 knees (31.4%) questionable candidates for UKA.

The current study suggests that a considerably lower percentage of Chinese patients meet the Oxford criteria for a UKA. Chinese patients presented later for surgery than patients from Malaysia and India because of increased fear of surgery and a lack of social support [25]. These factors might contribute to more severe disease at the time of surgery. While there might be cultural and socioeconomic reasons for patients seeking out care at later stages of the disease there might be also anatomic variances that trigger differences in the amount of deformity. The modified Keyes classification, which is described as a reliable tool for the assessment of ACL function [14], showed an interesting finding in the current study. Only one patient showed evidence of an ACL deficient wear pattern. ACL deficiency therefore appears to be rather uncommon in the Chinese population.

Computer navigation for UKA leads to an improvement in implant position with fewer outliners [26]. Despite improvement in mechanical axis restoration, long-term survival was comparable to standard implantation techniques [27]. In a recent metaanalysis an advantage in clinical or long-term follow could not be established in favor of navigated UKA [28]. Recently Blyth *et al*. [29] reported for robotic assisted UKA superior results in the early postoperative clinical outcome, however one-year results were equal with favorable results in patients with higher activity levels.

## CONCLUSION

A lower percentage of the current Chinese patient population meets the indication criteria for UKA. While Chinese patients have more severe deformity and reduced range of motion, ACL deficiency is a rare contraindication for UKA in China. Data of the current study supports that UKA is a valuable option for selected patients in China, however, considering the smaller number of patients that meet the indication criteria it might be beneficial to concentrate this surgery in specialized centers.
